# Discovery of a non-cationic cell penetrating peptide derived from membrane-interacting human proteins and its potential as a protein delivery carrier

**DOI:** 10.1038/srep11719

**Published:** 2015-06-26

**Authors:** Hyo Young Kim, Soo Young Yum, Goo Jang, Dae-Ro Ahn

**Affiliations:** 1 Center for Theragnosis, Biomedical Research Institute, Korea Institute of Science and Technology (KIST), Hwarangno 14-gil 5, Seongbuk-gu, Seoul 136-791, Republic of Korea; 2 Department of Biological Chemistry, Korea University of Science and Technology (UST), KIST campus, Republic of Korea; 3 Laboratory of Theriogenology, Department of Veterinary Clinical Science, College of Veterinary Medicine and BK21 PLUS Program for Creative Veterinary Science Research, Seoul National University; 4 Emergence Center for Food-Medicine Personalized Therapy System, Advanced Institutes of Convergence Technology, Seoul National University, Gyeonggi-do 443-270, Korea

## Abstract

Cell penetrating peptides (CPPs) are peptides that can be translocated into cells and used as a carrier platform for the intracellular uptake of cargo molecules. Subject to the source of CPP sequences and their positively charged nature, the cytotoxicity and immunogenicity of conventional CPPs needs to be optimized to expand their utility for biomedical applications. In addition to these safety issues, the stability of CPPs needs to be addressed since their positively charged residues are prone to interact with the biological milieu. As an effort to overcome these limitations of the current CPP technology, we isolated CPP candidate sequences and synthesized peptides from twelve isoforms of annexin, a family of membrane-interacting human proteins. The candidate screen returned a CPP rich in hydrophobic residues that showed more efficient cellular uptake than TAT-CPP. We then investigated the uptake mechanism, subcellular localization, and biophysical properties of the newly found CPP, verifying low cytotoxicity, long-term serum stability, and non-immunogenicity. Finally, model proteins conjugated to this peptide were successfully delivered into mammalian cells both *in vitro* and *in vivo*, indicating a potential use of the peptide as a carrier for the delivery of macromolecular cargos.

Cell penetrating peptides (CPPs), also called protein transduction domains (PTDs), have been numerously identified and established for the *in vitro* and *in vivo* delivery of bioactive cargos since the discovery of the first CPP, the TAT peptide of human immunodeficiency virus (HIV) in 1988^1^. Currently, there are five classes of known CPPs, categorized by the source of their sequences: 1) cationic CPPs derived from heparin-, RNA- and DNA-binding proteins^2^,^3^,^4^, 2) hydrophobic or amphiphilic CPPs derived from signal peptides, often combined with nuclear localization signals (NLS)^5^,^6^, 3) CPPs derived from antimicrobial peptides containing proline-rich sequences^7^,^8^, 4) CPPs derived from proteins of microorganisms, such as Vpr of HIV-1^9^ and Inv3 from a *Mycobacterium tuberculosis* membrane protein^10^, and 5) CPPs derived from phage and plasmid display screenings. Among them, there is a limited number of hydrophobic CPPs^11^, whereas many cationic and amphipathic CPPs have been discovered^12^. Although the potential of CPPs for molecular delivery seems promising, it is tempered with several limitations such as poor serum stability and considerable cytotoxicity. In addition to these issues of cationic peptides in general, CPPs recruited from a peptide library, or derived from microorganisms such as viruses and bacteria, feature potential risks of immunogenicity and toxicity^13^. These problems arising from CPP sequences of non-human origin can be circumvented by utilizing human protein sequences. Moreover, if the CPPs of human proteins are non-cationic, shortcomings of the conventional cationic CPPs can also be avoided.

In this context, we searched for CPP sequences in membrane-interacting human proteins containing membrane-binding sequences and assumed that their phospholipid-interacting part would be a good potential candidate for a CPP. Most membrane-interacting proteins such as G-proteins, protein kinases, hormones, antimicrobial peptides, and neurotoxins^14^ are either membrane-associated enzymes (e.g., signal peptidases), membrane-targeting domains (endosome- or Golgi-associated proteins)^15^, or structural domains for the attachment of proteins to the membrane, e.g., annexins^16^ and γ-carboxyglutamic acid (GLA)-rich domains^17^. Among them, we selected the calcium ion-regulated annexin proteins, which are membrane-associated, regulate the actin dynamics through their membrane interaction^18^, and bind to negatively charged head groups of phospholipids in the membrane^19^. In eukaryotic cells, annexin controls the intracellular calcium ion level by responding to extracellular stimulation, implicating that the protein has versatile interactions inside as well as outside of the membrane (Fig. 1). Indeed, annexin II interacts with phosphatidylinositol 4,5-bisphosphate (PI(4,5)P_2_)^20^, while annexin III, IV, V, and VI bind to phosphatidylethanolamine (PE) found in the cytoplasmic leaflet of the lipid bilayer^16^ . This led us to hypothesize that the annexin family proteins are capable of penetrating through the cell membrane and thus contain a CPP part. To verify this hypothesis, we selected several CPP candidate sequences from annexin proteins and estimated the cellular uptake efficiency of these peptides. For the most potent cell penetrating peptide, we further investigated biophysical properties such as uptake mechanism, cytotoxicity, immunogenicity, serum stability, and subcellular localization. After substantiating the properties of the newly found CPP, we examined the delivery of protein cargos such as β-galactosidase and Cre recombinase using the annexin-derived CPP to evaluate the peptide’s utility for delivering functional macromolecules. Finally, we evaluated the delivery efficiency of the cargo protein by the CPP *in vivo*.

## Results

### Design and preparation of CPP candidates

**Figure 1:**
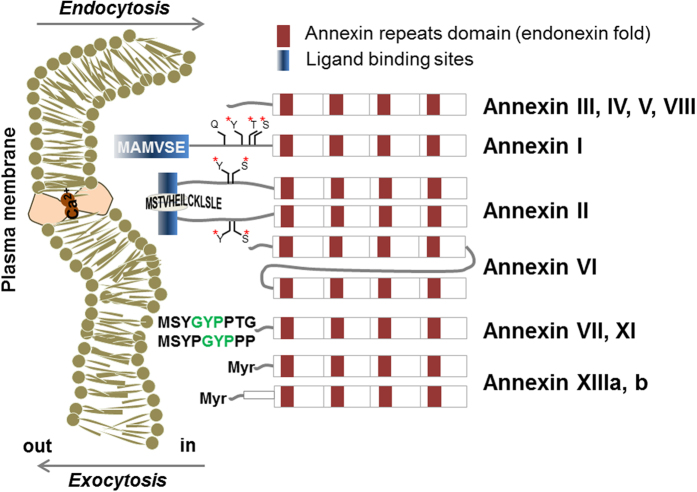
Schematic presentation of annexin isoforms and their N-terminus interacting with plasma membrane Annexin proteins has 12 types in a human form. Structures of annexins are consists of 4–8 annexin repeats domain that has the endonexin fold (red blocks). Annexin III, IV, V, and VIII has a simple structure having 12–19 residues length of N-terminal domains while annexin XI has a longer length. Annexin I and II have amphipathic α-helices in N-terminus, with phosphorylation sites for serine, threonine, and tyrosine. A glutamine residue is a linking site of a transglutaminase at position 18 in annexin I. Annexin VII and XI has the long N-terminal domain which is composed with glycine, tyrosine, and proline residues (green letters). Annexin XIII is different from other annexins as it has a myristoylation (Myr) of N-terminal domain. All annexins can be transported in and out of plasma membrane via endocytosis or exocytosis with/without calcium ion. This figure describes the endogenous annexins, which has the interaction with extracellular membrane, as proven from the experiment using artificial membrane, such as multivesicular bodies (MVBs)^49^.

We designed CPP candidate sequences from cell penetrating moieties representing the membrane-interacting domains of the endogenous annexin family^21^ (Fig. 1). The N-terminal regions of twelve annexin A isoforms (AA1H to AA13H in Table 1) were chosen as CPP candidates since the residues in this region are known to have calcium-sensitive lipid-binding or phospholipid-binding properties^15^. Interestingly, N-terminus sequences of annexin proteins are composed of hydrophobic residues with a charge of 0 to 1, unlike another well-known CPP, TAT, that has eight charged residues of arginine (Table 1). The twelve CPP candidates were synthesized with a fluorescent label by using the standard Fmoc-based solid phase peptide synthesis protocol. All synthesized peptides were characterized by MALDI-TOF mass analysis (Table 1).

### Cellular uptake of annexin A-derived CPPs

**Figure 2:**
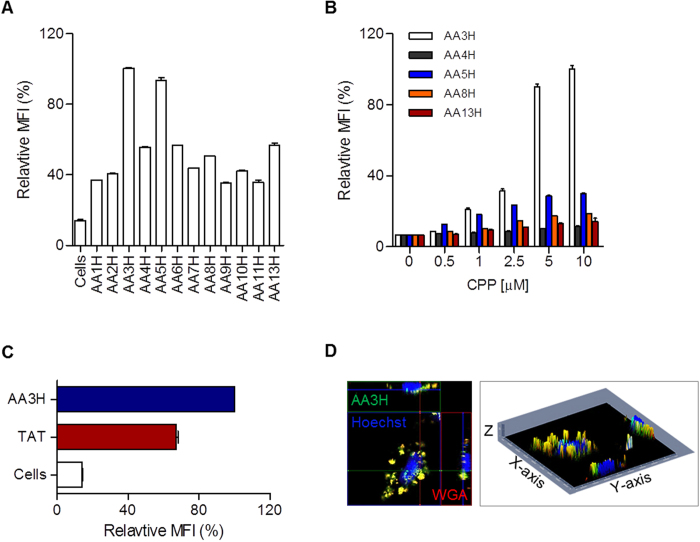
Cellular uptake of CPP candidates derived from annexin isoforms. (**A**) Mean fluorescence intensity (MFI) of the cells treated with fluorescently labeled CPP candidates (1 μM) was determined relative to the highest intensity with AA3H. (**B**) Dose-dependent uptake efficiency of the selected five CPP candidates such as AA3H (white bars), AA4H (gray bars), AA5H (blue bars), AA8H (orange bars), and AA13H (red bars) determined at various concentrations (0, 0.5, 1, 2.5, 5, and 10 μM). (**C**) Comparison of cellular uptake efficiency between AA3H and TAT. In all cytometric analysis, each bar represents the average of three independent experiments. (**D**) An ortho-view of z-stack images of a cell to confirm intracellular delivery of AA3H. The horizontally sectioned side view by the green line and the vertically sectioned side view by the red line were displayed at the top and the right regions, respectively, in the fluorescence microscopic image (left). Plasma membrane and nucleus were visualized by staining with Alexa Fluor 594-conjugated wheat germ agglutinin (WGA) (red) and a Hoechst dye (blue). A z-stacked image indicates clearly intracellular delivery of AA3H as observed either in the cytoplasm (green color) or in the plasma membrane (yellow color due to superimposition with the red color, right).

The cellular uptake efficiency of the twelve candidate peptides into HeLa cells was tested using a flow cytometer (Fig. 2A). Five candidates (AA3H, AA4H, AA5H, AA8H, and AA13H) were found to have cell-penetrating properties with a significant cellular uptake efficiency at 1 μM. On further examination at various concentrations, dose-dependent cellular uptake efficiency and distinguished potency in intracellular translocation in the order of AA3H > AA5H > AA8H > AA13H > AA4H were established (Fig. 2B). When the uptake efficiency of the most potent one, AA3H, was compared with that of the widely used conventional CPP TAT peptide (Fig. 2C), AA3H was delivered into the cells better than TAT. In contrast, when compared with the fully cationic R9 peptide, AA3H showed much lower cellular uptake (Supplementary Fig. S1A). For assessing uptake of AA3H by confocal microscopy, HeLa cells were treated with AA3H and fluorescently analyzed. Z-stack images (30 sections) of selected cells were obtained (Supplementary Fig. S2) and constituted into a 2.5D image of the cells. Fig. 2D clearly shows that AA3H (green color) resides inside the cell membrane (wheat germ agglutinin, red color), thereby confirming that the cytometry profiles obtained were not due to adsorption on the cell surface but to intracellular delivery of the peptide.

### Cellular uptake mechanism of AA3H

**Figure 3:**
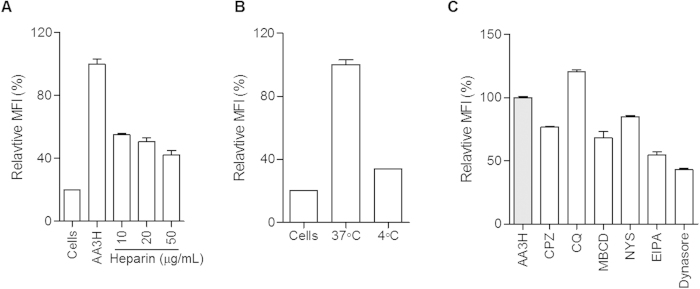
Cellular internalization mechanisms of AA3H. (**A**) Influence of heparin on intracellular delivery of AA3H analyzed using a flow cytometer. Each bar represents the average of three independent experiments and expressed as a percentage value relative to the delivery level of AA3H in the absence of heparin. (**B**) Energy dependent penetration of AA3H. The relative penetration efficiency at the lowered temperature (4 °C, middle) was compared with that at 37 °C (right) (**C**) Delivery of AA3H into the cells pre-treated with endocytosis inhibitors. The delivery level of AA3H into untreated cells was used as the control and displayed as the gray bar. Each bar represents the average of three independent experiments.

After settling on AA3H as the best CPP candidate, we investigated the uptake mechanism of the peptide by employing several well-established endocytosis inhibitors^22^ because the internalization into mammalian cells of biomacromolecules such as peptides and proteins is mainly accomplished through endocytic mechanisms^23^. We first tested whether the cellular uptake of AA3H was affected by heparan sulfate proteoglycan (HSPG), a component involved in the initial step of membrane-CPP interaction^24^. After treatment with several concentrations of heparin sulfate for 30 min, HeLa cells were incubated with AA3H for 4 h. As shown in Fig. 3A, the intracellular delivery of the peptide decreased by 45% after treatment with 50 μg/mL heparin sulfate, which was expected since most CPPs penetrate the cell membrane *via* HSPG-mediated pathways. Despite the presence of only one arginine in the AA3H sequence, the peptide still appeared to interact with HSPG during the initial step of penetration (Fig. 3A), probably due to a contribution by tryptophan, with the basic residue triggering glycosaminoglycan (GAG)-dependent endocytosis^25^. However, concentrations of heparin higher than 50 μg/mL did not lead to an additional inhibition of peptide delivery, suggesting that the uptake may also be driven by interactions with other membrane components such as phospholipids. The phospholipids known to interact with annexin III, the parent protein of AA3H, were found to also bind AA3H (Supplementary Fig. S3)^26^. This indicates that the binding preference of the protein is preserved in the peptide, probably contributing to the cell-penetrating properties of the peptide.

Subsequently, we examined the effect of lowered temperature on the cellular penetration to test whether the translocation of the peptide is mediated via an energy-dependent pathway^27^. AA3H was incubated with HeLa cells at 4 °C for 2 h, and cellular uptake efficiency was analyzed by flow cytometry. As shown in Fig 3B, the cellular uptake of AA3H-CPP at 4 °C was approximately 66% lower than that at 37 °C (control), indicating that the peptide was internalized into cells via an energy-dependent endocytosis mechanism.

To examine the details of the endocytosis mechanism, cells were pre-incubated with several inhibitors of distinct aspects of the endocytosis mechanism before treatment with AA3H (Fig. 3C). A 30-min pre-treatment of cells with chlorpromazine (CPZ), an inhibitor of clathrin-dependent endocytosis^28^, resulted in a 23.5% decrease in AA3H-CPP uptake. When chloroquine (CQ), a disruptor of endosome/lysosome, was used instead, a 20.5% increase in mean fluorescence intensity was observed. However, this was not due to an increase of uptake but because of the dequenched intensity of fluorescein after escape from the acidic environment of the endosome, because pH-insensitive dye-labeled AA3H did not show any increased intensity upon treatment with CQ (Supplementary Fig. S4). Treatment with methyl-β-cyclodextrin (MβCD) and nystatin (NYS), related to the caveolae-dependent mechanism, significantly reduced the uptake of the peptide by 32% and 15%, respectively. In an experiment to test the dynamin dependency of AA3H-CPP penetration by employing the specific inhibitor of dynamin, dynasore, a 57% decrease of the uptake was observed. A significant inhibition of peptide delivery was also obtained by treatment with 5-(N-ethyl-N-isopropyl) amiloride (EIPA), an inhibitor of macropinocytosis. These results indicate that the uptake of AA3H is not accomplished via a single mechanism but via a complex combination of various processes such as clathrin-mediated, caveolae-mediated endocytosis mechanisms and macropinocytosis.

### Subcellular localization of AA3H

**Figure 4:**
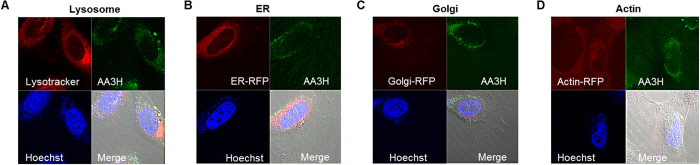
Subcellular localization of AA3H. Subcellular localization of AA3H in various intracellular organelles such as (**A**) lysosome, (**B**) ER, (**C**) Golgi, and (**D**) actin were examined by using the organelle-specific red-staining reagents. The localization was estimated from the presence of the orange color resulting from superimposition of the red color by the staining reagents and the green color by the peptide.

Previously, many CPPs delivered by endocytosis have been generally destined for the lysosome for degradation, while several CPPs have been able to escape^29^. In particular, CPPs utilizing the caveolae-mediated pathway have been transferred into the Golgi apparatus or endoplasmic reticulum (ER) after invagination of the plasma membrane by dynamin action^29^. To see whether AA3H follows similar localization patterns, we attempted to trace the subcellular localization of the internalized peptide by confocal microscopy using organelle-specific fluorescent staining reagents. As shown in Fig. 4A, a certain amount of AA3H peptide was found in lysosomes (stained with Lysotracker), but not in the nucleus (stained with a Hoechst dye), indicating that the peptide may undergo partial decomposition in the lysosome. These results could explain the reduced accumulation of AA3H-CPP in lysosomes in the presence of an endosome/lysosome disrupter, as observed during flow cytometry analysis (Fig. 3C). However, no peptide was found in the ER and the Golgi when examined for localization in these subcellular organs using organ-specific binders labeled with a fluorescent protein (Fig. 4B,C). As membrane ruffling by actin rearrangement is required for the process of macropinocytosis, we tested for possible interactions of AA3H with actin and found partial colocalization in the cells (Fig. 4D). Overall, these results suggest that after caveolae-mediated endocytosis, the peptide seems to escape before entering ER and Golgi and is found in the cytoplasm. While AA3H does not enter the nucleus, a minor fraction of CPPs is found in the lysosome and plasma membrane, as inferred by the membrane-associating properties of annexin proteins^19^. In addition, our results are consistent with the previous finding that the hydrophobicity of a peptide improves its escape rate from endosomes^30^. The hydrophobic nature of AA3H may facilitate its uptake into the cytoplasm and the destabilization of the endosomal membrane for enhanced cytoplasmic release.

### Conformation of AA3H

**Figure 5:**
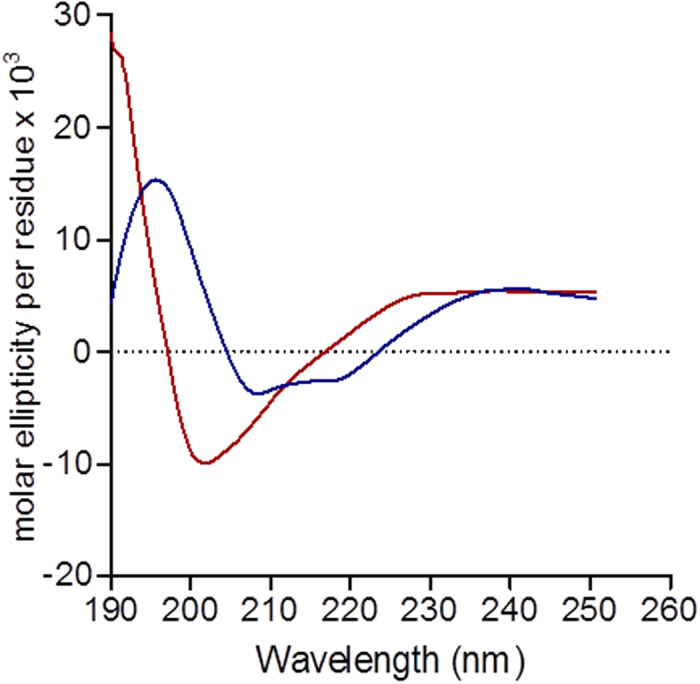
CD spectra of peptides. The CD spectra of AA3H (blue) and TAT (red) solutions (0.1 mg/mL) in phosphate buffer (pH 6.4) measured at 20 °C.

After studying cellular uptake properties of AA3H, we investigated the conformational state of AA3H. CD spectra of AA3H revealed random coil and β-sheet structures. The profile shows a typical peak at around 196 nm as a positive band of β-sheet structure (Fig. 5, blue line). In contrast, the CD spectra of TAT showed a typical random coil structure with a strong negative band at 200 nm, supporting a previous report^31^ (Fig. 5, red line). The CD profiles were similar at various concentrations of peptides, suggesting that there was no aggregation or dimerization. The conformation of the peptide is rather unusual since, according to previous reports^32^, most amphipathic CPPs show α-helical structures.

### Analysis of serum stability of AA3H

**Figure 6:**
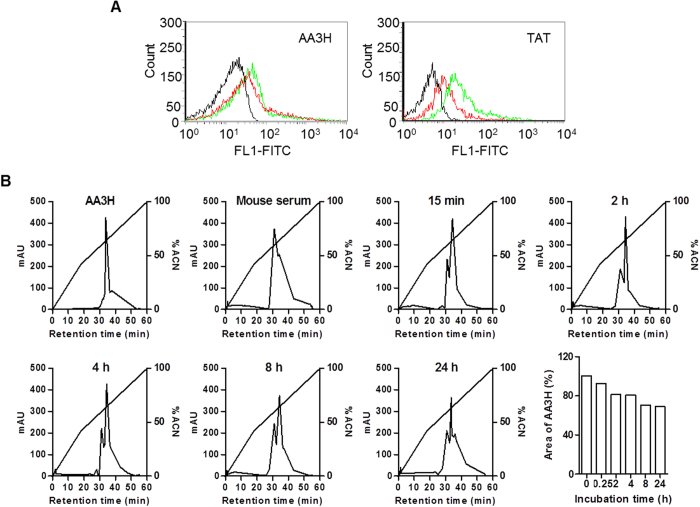
Serum stability of AA3H. (**A**) The HeLa cells treated with AA3H (left) or TAT (right) peptides (1 μM) in the presence (red trace) or absence (green trace) of 10% mouse serum were analyzed by a flow cytometer. The black profile indicates the untreated control cells. (**B**) AA3H was incubated with 10% mouse serum for several time points (0, 15 min, 2, 4, 8, 24 h) at 37 °C. The AA3H peak was appeared at 34.26 min and the peak of serum was 31.01 min. The relative peak area (%) of AA3H at each time point was calculated based on the initial peak area and displayed in a bar chart (right bottom).

Serum stability is an important precondition for applications of CPPs as delivery carriers *in vitro* and *in vivo*. The serum stability of AA3H was analyzed by determining the half-life of the peptide in mouse serum. Whereas TAT showed significantly lowered efficiency in the presence of serum (Fig. 6A, right), the delivery efficiency of AA3H was not considerably affected by the presence of serum (Fig. 6A, left). AA3H was incubated in serum solution at 37 °C, and the reaction was analyzed at various time points using reverse-phase HPLC for a time-course study of peptide degradation. Although AA3H showed gradual degradation over the incubation time, ca. 70% of AA3H was still undamaged after 24 h (Fig. 6B), displaying much higher serum stability than other known CPPs^33^. This is consistent with the observation that cellular uptake of AA3H was not reduced in serum-containing medium (Fig. 6A).

### Analysis of cellular toxicity and immunogenicity of AA3H-CPP

**Figure 7:**
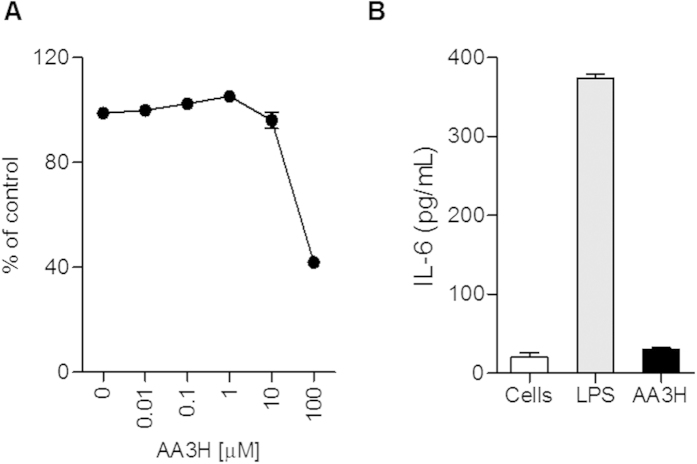
Toxicity and immunogenicity test for AA3H. (**A**) Cell viability 24 h after treatment of AA3H peptide at varying concentrations (0, 0.01, 0.1, 1, 10, 100 μM). Each point represents the average of four independent experiments. (**B**) The level of IL-6 released from Raw 264.7 cells was determined after treatment of AA3H (10 μM, black bar) or LPS (10 μg/mL, gray bar). The level from untreated cells is displayed with a white bar. Each bar represents the average of three independent experiments.

Subject to size and sequence of CPPs, peptides can exhibit cytotoxicity and immunogenicity as potential risks for their biomedical applications. In order to evaluate the potential toxicity of the peptide, HeLa cells were treated with 0.01–100 μM AA3H for 24 h, and cell viability was determined by a cell-counting assay. The peptide affected the cell viability only negligibly at 10 μM, indicating that AA3H is non-cytotoxic in the concentration range used for the cellular uptake experiments (Fig. 7A).

To assess the potential immunogenicity of AA3H, we treated macrophage cells with the peptide and quantified the cellular release of interleukin-6 (IL-6), which is secreted by macrophages and T cells to stimulate immune responses in an acute phase and the innate immune system^34^. After incubation of Raw 264.7 cells in the presence of 10 μM AA3H for 24 h, the amount of IL-6 released into the culture medium was estimated using an ELISA kit and compared to the amount obtained from lipopolysaccharide (LPS)-treated cells as a positive control (Fig. 7B). While IL-6 secreted in response to LPS as a standard immunogenic material was determined as 373 pg/mL, AA3H-stimulated cells released 30 pg/mL of IL-6, similar to non-treated control cells (20 pg/mL), indicating that AA3H is a virtually non-immunogenic peptide. Therefore, this CPP shows a good potential as a safe carrier system in various *in vivo* applications.

### Intracellular delivery of functional proteins using AA3H as a carrier

**Figure 8:**
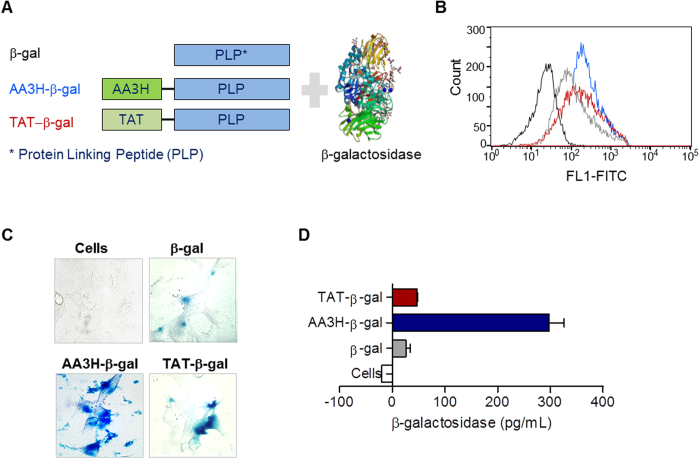
Intracellular delivery of β-galactosidase using CPPs and the activity of the delivered enzyme. (**A**) Constructs of CPPs conjugated with the protein linking peptide (PLP) to test the delivery ability of β-galactosidase protein by CPPs into cells. (**B**) The cells treated with AA3H-PLP (blue trace), TAT-PLP (red trace), or PLP (gray trace) were analyzed on a flow cytometer to estimate intracellular delivery of the peptides. The black trace indicates the untreated cells. (**C**) Microscopic images of cells treated with the AA3H-β-galactosidase conjugate (bottom left), the TAT-β-galactosidase conjugate (bottom right), or PLP-β-galactosidase conjugate (top right). The image of untreated cells was presented at top left. (D) Quantitative analysis of the activity of intracellularly β-galactosidase by AA3H (blue bar), TAT (red bar), and PLP (gray bar). Activity from the untreated cells was shown with a white bar. Each bar represents the average of three independent experiments.

To test the potential of AA3H as a carrier for protein delivery, the peptide was conjugated with two different functional proteins such as β-galactosidase and Cre recombinase, active in cytoplasm and nucleus, respectively. For conjugation, the transglutaminase reaction was used as previously reported, connecting the acyl group of glutamine residues in peptides with the amino group of lysine residues in proteins to form a γ-glutamyl-ε-lysine structure^35^. For enhanced efficiency of the conjugation reaction, a protein linking peptide (PLP) containing multiple arginine residues was incorporated into the AA3H sequence (Fig. 8A). The cellular uptake of PLP-conjugated CPPs was initially assessed by flow cytometry utilizing the fluorescence label of the peptide. PLP alone could also be delivered, probably because of multiple arginine residues in the linker, although the uptake efficiency was not as high as with AA3H conjugation (Fig. 8B). When cells treated with AA3H-conjugated β-galactosidase were incubated with the X-gal substrate 5-bromo-3-indolyl β-D-galactopyranoside, the blue color of the hydrolysis product generated by intracellularly delivered β-galactosidase was observed in cells under microscopic analysis and thus clearly indicated that the enzyme was successfully delivered into the cells (Fig. 8C, AA3H-β-gal). In contrast, enzyme conjugated with PLP alone did not show considerable activity due to inefficient delivery of the protein (Fig. 8C, β-gal). Even TAT-conjugated enzyme was not delivered as efficiently as the AA3H-conjugated one, producing a relatively smaller amount of the colored product (Fig. 8C, TAT-β-gal). Uptake of β-galactosidase was additionally confirmed by measuring enzyme activity with an assay using the chromogenic substrate *o*-nitrophenyl-β-galactoside and reading absorbance at 405 nm. In this assay, β-galactosidase delivered by AA3H produced 298 pg/mL of *o*-nitrophenol, a 6-fold higher amount than obtained with the enzyme delivered by TAT (47 pg/mL) (Fig. 8D).

**Figure 9:**
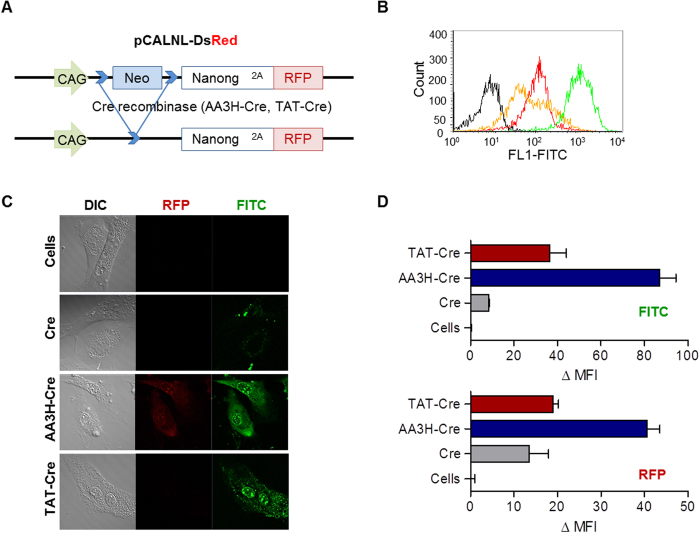
Intracellular delivery of Cre-recombinase using CPPs and the activity of the delivered enzyme. (**A**) Constructs of pCALNL-DsRed fibroblast cells to express RFP by cleaved loxP site. Two loxP sites indicated bright blue that holding RFP expression. During recombination by Cre, a stop codon with Neo is removed to lead continuous transcription through RFP gene. (**B**) Uptake of AA3H-PLP (green trace), TAT-PLP (red trace), and PLP (orange trace) into primary fibroblast cells was analyzed on a flow cytometry. The black trace indicates untreated cells. (**C**) Confocal microscopic images of cells treated with AA3H-Cre conjugate (third row), the TAT-Cre conjugate (second row), and the PLP-Cre conjugate (second row). Delivery of the conjugates (third column, FITC) and the RFP expression (second column, RFP) due to recombination by the delivered Cre conjugates was monitored. (**D**) Quantitative analysis of the delivery of the conjugates (top) and the RFP expression by the conjugates (bottom) performed on a flow cytometer. AA3H-Cre: blue bar, TAT-Cre: red bar, PLP-Cre: gray bar, untreated cells: white bar. Each bar represents the average of three independent experiments.

After demonstrating the feasibility of protein delivery based on AA3H, we attempted to deliver another functional protein, Cre recombinase (Cre), to confirm the utility of the peptide as a general carrier for the delivery of macromolecules. Cre is an enzyme that performs a recombination reaction between two specific recognition sites called loxP. To test intracellular activity of Cre, we prepared fibroblast cells containing plasmids that can be recombined by the enzyme to express red fluorescence protein (RFP) (Fig. 9A)^36^. After confirming uptake of CPPs into the fibroblasts (Fig. 9B), we prepared CPP-conjugated Cre using the same procedure as for CPP-conjugated β-galactosidase. After treatment of the cells with CPP-Cre conjugates, the expression level of RFP, as reflecting the activity of the enzyme, was monitored using confocal microscopy (Fig. 9C). Again, AA3H conjugation yielded higher RFP expression than TAT conjugation. When analyzed using a flow cytometer for quantitative comparison, CPP-Cre conjugates penetrated into primary fibroblast cells as well as in HeLa cells (Fig. 9D, top). Cells treated with AA3H-conjugated Cre exhibited a 2.1-times higher RFP expression level than cells treated with the TAT-conjugated enzyme (Fig. 9D, bottom).

### *In vivo* delivery of β-galactosidase by using AA3H as a carrier

**Figure 10:**
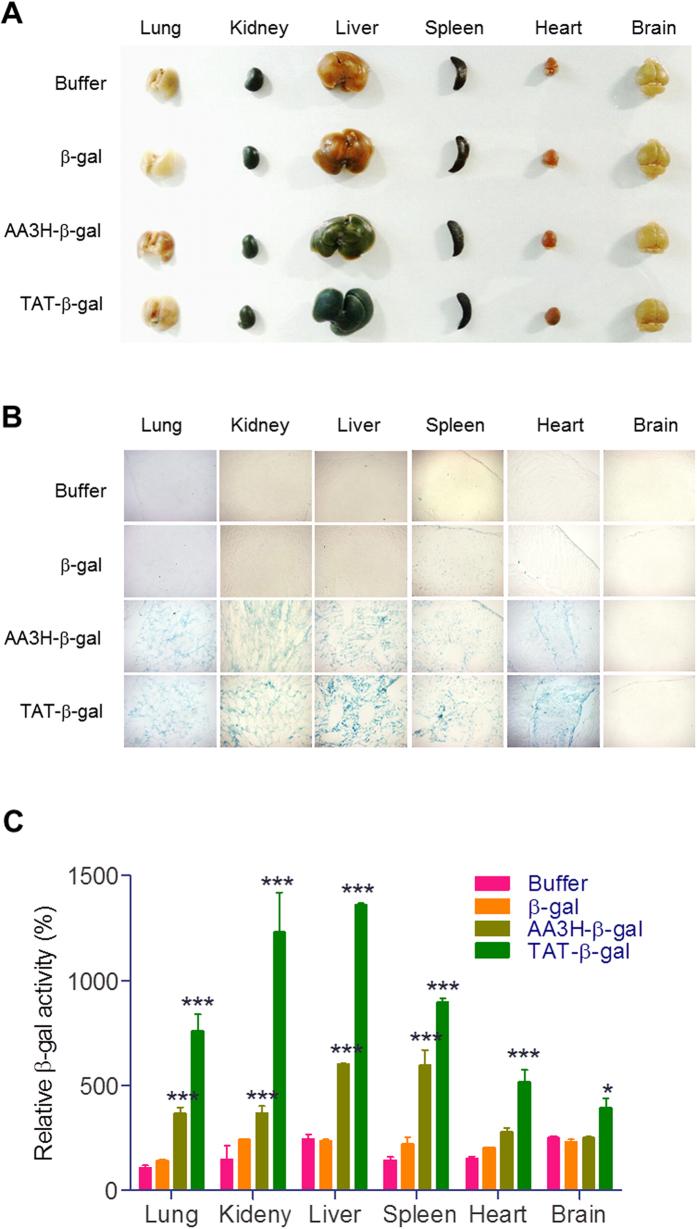
*In vivo* distribution of β-galactosidase using CPPs. (**A**) Harvested organs from mice injected intraperitoneally with the CPP-β-gal conjugates after staining with the X-gal substrate. (**B**) Sectioned images of each organ shown in (**A**). (**C**) Quantitative analysis of β-galactosidse activity in each tissue.

Finally, biodistribution of a cargo protein by AA3H was assessed to examine whether the carrier property of the peptide observed at the *in vitro* level could be reproduced *in vivo*. After intraperitoneal administration of AA3H-β-gal to mice, main organs were harvested and treated with X-gal to estimate the amount of the delivered enzyme in each organ. The enzyme was detected in main organs except the brain (Fig. 10A). Histological analysis of tissue sections using microscopy indicated successful *in vivo* delivery of the protein with AA3H as a carrier (Fig. 10B). However, when compared with TAT-β-gal quantitatively with enzyme assays for tissue lysates, AA3H-β-gal was delivered less than TAT-β-gal in all organs *in vivo*. This is possibly due to the much slower uptake kinetics of AA3H with ~2 h of *t*_*0.5*_ (half-time of internalization) compared to TAT with 1 min of *t*_0.5_^37^, which allows AA3H to be cleared by body fluid around tissues before translocation into cells (Fig. 10C). Therefore, the improved intracellular uptake efficiency of AA3H-β-gal compared to TAT-β-gal observed *in vitro* could not be reproduced under *in vivo* conditions. Nevertheless, these data qualify the CPP AA3H as a molecular carrier, demonstrating successful protein delivery based on AA3H and suggesting that our newly found CPP has a great potential to be used for delivery of various functional macromolecules *in vitro* and *in vivo*.

## Discussion

Since the first discovery of TAT, many CPPs were introduced as drug delivery platforms^38^, therapeutic agents^39^,^40^,^41^, and imaging probes *in vivo*^42^. These CPPs are either positively charged with multiple cationic residues or based on sequences of non-human organisms^43^,^44^,^45^. Membrane-interacting proteins can be considered a good potential source for discovery of new CPP sequences since membrane interaction is the initial step of the cell-penetrating process. Previously, a non-cationic CPP was obtained from a membrane protein of *Mycobacterium tuberculosis*^10^, while no CPPs have been identified from membrane-binding human proteins. To circumvent potential risks posed by sequences of non-human organisms such as bacteria and viruses, developing CPPs originated from human proteins is immensely important because CPPs with inherently human-friendly sequences would be preferable for practical use in the clinic.

AA3H showed higher serum stability than TAT, rendering it attractive for *in vivo* applications. Since proteolytic degradation of peptides in serum depends partly on their content of arginine and lysine residues^46^, the relatively hydrophobic AA3H peptide, containing only one arginine residue, could thus be a less preferred substrate for proteases than TAT, containing six arginine and two lysine residues. Whereas D-amino acids could be considered an alternative for preparation of CPPs to increase serum stability, such non-natural peptide backbones would be costly and may cause potential side-effects such as undesired immune responses^47^,^48^, making natural backbones more desirable for biomedical applications.

AA3H was a far better carrier than TAT for intracellular delivery of functional proteins *in vitro*. This unexpected difference may not have just been caused by a higher penetrating ability of AA3H but also by a difference in efficiency of the conjugation reaction. In contrast to a lack of lysine in the AA3H sequence, the TAT sequence contains both lysine and arginine residues required for conjugation by transglutaminase, thereby possibly yielding undesired self-conjugated products. The lack of lysine in AA3H is particularly advantageous if CPP conjugation with cargo proteins is performed using transpeptidase rather than cloning and expression of CPP-fused protein. When we used CPP-protein conjugates purified by removing peptides and possible TAT dimers, we could still observe higher protein delivery efficiency by AA3H compared to TAT (Supplementary Fig. S5), confirming that AA3H is more effective than TAT as a vehicle for intracellular protein delivery. Although *in vivo* delivery efficiency by these CPPs was not identical to that observed at the *in vitro* level, successful biodistribution of AA3H-conjugated protein clearly illustrates that this peptide could be utilized not only for *in vitro* but also for *in vivo* applications.

In summary, we have designed CPP candidate sequences from N-terminal sequences of annexin, a human protein family. After synthesis of the sequences, we found a novel CPP named AA3H, composed of hydrophobic sequences recruited from a membrane-interacting human protein, showing excellent cell penetrating ability. Cellular uptake of the peptide was mediated by various endocytosis mechanisms. The peptide was localized in the cytoplasm but not in the nucleus. According to CD spectra, the peptide showed a β-sheet conformation, which is unlike that of previously known amphipathic CPPs. The biophysical properties of the new peptide such as high serum stability, non-immunogenicity, and non-cytotoxicity appear beneficial for biomedical applications. Utility of the CPP as a carrier for biomacromolecules has been successfully demonstrated by the delivery of two functional proteins, β-galactosidase and Cre recombinase, both *in vitro* and *in vivo*. We therefore expect the newly found CPP AA3H to provide a useful delivery platform for various *in vitro* and *in vivo* applications.

## Methods

### Reagents

For peptide synthesis, all chemicals including Rink amide resin, Fmoc-protected amino acids, 1-hydroxybenzotriazole (HOBt), and *O*-benzotriazole-*N,N,N’,N’*-tetramethyl-uronium-hexafluoro-phosphate (HBTU) were purchased from Novabiochem (Darmstadt, Germany). Fluorescein isothiocyanate (FITC), *N,N*-diisopropylethylamine (DIPEA), dimethylformamide (DMF), piperidine, trifluoroacetic acid (TFA), triisopropylsilane (TIS), acetonitrile, and the endocytosis inhibitors chlorpromazine hydrochloride (CPZ), 5-(N-ethyl-N-isopropyl)-amiloride (EIPA), dynasore, methyl-β-cyclodextrin (MβCD), and chloroquine (CQ) were from Sigma Aldrich (St. Louis, MO, USA). The wheat germ agglutinin conjugate (WGA Alexa Fluor 594 conjugate), actin-RFP, ER-RFP, and Golgi-RFP were from Molecular Probes (Carlsbad, CA, USA).

### Peptide synthesis

Peptides were synthesized at a 25-μmol scale using the standard Fmoc-based solid-phase peptide synthesis on Rink amide resin (0.7 mmol/g). For coupling reactions, Fmoc-protected amino acids (5 equivalents), HOBt (5 equivalents), HBTU (5 equivalents), and DIPEA (5 equivalents) in DMF were added to the solution of resin in DMF (0.5 mL). Deprotection of Fmoc was achieved by incubation of the resin with 20% (v/v) piperidine in DMF (0.5 mL) at room temperature for 30 min. After N-terminal FITC labeling, peptides were deprotected and cleaved from resin by incubation in deprotection solution (1 mL; 95% (v/v) TFA, 2.5% TIS, and 2.5% H_2_O) at room temperature for 2 h. Peptides were precipitated by addition of diethyl ether (5 mL), centrifuged at 1,500 × *g* for 5 min, and purified on C18 reversed-phase (RP) HPLC (Agilent Technologies, Santa Clara, CA, USA) using acetonitrile and water containing 0.1% TFA as eluents. All purified peptides were characterized using MALDI-TOF mass spectrometry (Kangwon National University, Kangwon, Korea).

### Cells and cell culture

Human cervical carcinoma-derived HeLa cells were obtained from American Type Culture Collection (ATCC) and maintained in DMEM supplemented with 10% fetal bovine serum (FBS), 1% penicillin, and 1% streptomycin (Gibco, Carlsbad, CA, USA) at 37 °C with 5% CO_2_ in a humidified incubator. Murine macrophage cells (Raw 264.7) were cultured under identical conditions. pCALNL-DsRed cells were prepared as previously described^36^ and maintained in DMEM with 10% FBS.

### Cellular uptake and localization of CPPs

For assessing the uptake of CPP candidates by HeLa cells, cells were seeded onto 6-well plates (1.0 × 10^6^ cells/well). After 24 h, cells were washed two times with serum-free DMEM and incubated with CPPs at indicated concentrations (0.1, 1, 10 μM) in serum-containing DMEM at 37 °C. Cells were washed two times with phosphate-buffered saline (PBS), incubated in trypsin-EDTA solution (0.01% trypsin) at 37 °C for 10 min to digest extracellular proteins, washed two times with ice-cold PBS, and finally resuspended in 500 μL of ice-cold PBS for flow cytometry assays. For endocytosis inhibitor treatments, cells were pre-incubated with various inhibitors (50 μg heparin, 50 μM CPZ, 100 μM CQ, 1 mM MβCD, 30 μg dynasore, or 50 μM EIPA) in serum-containing medium for 30 min before AA3H (1 μM) was added. The sample for flow cytometry analysis was prepared as described above. For the analysis of peptide internalization, a GUAVA flow cytometry system (Merck Millipore, Darmstadt, Germany) with the FL-1 channel for measuring fluorescence in GuavaSoft 2.6 was used according to the manufacturer’s instructions. Results were presented as a relative percentage value of untreated HeLa cells displayed as mean ± standard deviation (SD).

To visualize the cellular uptake of a peptide using confocal fluorescence microscopy, HeLa cells were seeded onto confocal bottom dish 35-mm plates (SPL, Gyeonggi-do, Korea) and incubated with AA3H (1 μM) for 4 h. Lysosomes and nuclei of AA3H-treated cells were stained with LysoTracker (Life Technologies, Carlsbad, CA, USA; 1 μg/mL for 30 min) and Hoechst (1 μg/mL for 5 min), respectively. For staining ER and Golgi, CellLight Reagents BacMam 2.0 (Life Technologies, Carlsbad, CA, USA) were used. Cells were cultured in confocal dishes for 24 h, transfected with red fluorescence protein (RFP) for 24 h, and treated with AA3H (1 μM) for 4 h. After washing two times with PBS, the intracellular fluorescence of HeLa cells was imaged using confocal laser scanning microscopy (LSM 700, Carl Zeiss, Jena, Germany).

### Circular dichroism (CD) spectroscopic measurements

CD spectra were recorded at 20 °C using a Jasco J-715 spectrometer (JASCO, Great Dunmow, UK) equipped with a PFD-350S temperature controller. All experimental samples were measured in a 1-cm quartz cell using a 260-190 nm measurement range, 100 nm/min scanning speed, 0.2 nm pitch, 4 s response time, and 1.0 nm bandwidth. The baseline was established with phosphate buffer, recorded under the same conditions, and subtracted automatically. For measurements, peptides were diluted to a final concentration of 0.1 mg/mL in 10 mM phosphate buffer (pH 6.4). Every sample was measured eight times, and cuvettes were washed three times with Milli-Q deionized H_2_O before each measurement.

### Serum stability assay

For intracellular uptake of CPPs in the presence of serum, cells were treated with peptides (1 μM) with or without 10% serum (Gibco, Carlsbad, CA, USA) for 4 h and analyzed by flow cytometry. Samples were prepared as described for cellular uptake above. AA3H (10 μM) was incubated in a serum solution at 37 °C for various periods (30 min, 2 h, 4 h, 8 h, and 24 h), and the mixture was analyzed by RP-HPLC using a C18 column (Agilent Technologies, Santa Clara, CA, USA).

### Cell viability assay

Cell viability was assessed using the Cell Counting Kit 8 (CCK-8; Dojindo, Kumamoto, Japan). HeLa cells were seeded into a 96-well plate, cultured until 80% confluence, and treated with AA3H (0 to 100 μM) for 24 h in DMEM containing 10% serum. WST-1 reagent (10 μL) was added to each well, followed by incubation for 2 h. The absorbance of each well was determined at 450–650 nm using a microplate reader system (Molecular Devices, Sunnyvale, CA, USA). Cell viability was presented as a relative percentage value of untreated HeLa cells displayed as mean ± standard deviation (SD).

### Immunogenicity assay

Raw 264.7 cells were seeded into 24-well plates and cultured for 24 h. Cells were grown to 80% confluence and treated with lipopolysaccharides (LPS, 10 μg/mL) as a positive control. After incubation of AA3H peptide (10 μM) with cells for 24 h, supernatants were transferred to 1.5 mL tubes and centrifuged at 10,000 × *g* for 10 min at 4 °C. For the detection of interleukin-6 (IL-6) a mouse IL-6 ELISA kit (Thermo Scientific, Waltham, MA, USA) was used. Results from triplicate experiments were analyzed by Prism software (GraphPad, La Jolla, CA, USA). Data are presented as mean ± standard deviation (SD).

### *In vitro* delivery of β-galactosidase by CPPs

Each CPP-PLP was conjugated with β-galactosidase (5 mg/mL in DMF, Sigma Aldrich) using transglutaminase (1 mg/mL, Sigma Aldrich) in conjugation buffer (50 mM Tris-HCl pH7.4, 5 mM CaCl_2_, 1 mM DTT) for 1 h at 37 °C. Cells were washed twice with serum-free DMEM and incubated in a mixture of serum-free medium (400 μL), the resulting conjugate solution (100 μL) at 37 °C for 6 h, washed thrice with serum-free DMEM and analyzed using flow cytometry and confocal laser scanning microscopy at 37 °C for 3 h. For microscopic analysis, cells were fixed in 1× fixation buffer (2% formaldehyde and 0.2% glutaraldehyde in PBS) for 10 min and washed twice with PBS. Using a β-galactosidase staining kit (GALS, Sigma Aldrich, St. Louis, MO, USA), cells were incubated with X-gal substrate at 37 °C for 2 h and washed twice with PBS. Stained cells were analyzed using a Nuance microscope (Nuance, Olympus; 100 × oil immersions). To quantify the activity of delivered β-galactosidase, cells containing CPP-β-galactosidase conjugates were treated as described above, except using *O*-nitrophenyl-β-galactoside (ONPG, Sigma Aldrich, St. Louis, MO, USA) as the substrate instead of X-gal. Cells were lysed with RIPA lysis buffer at room temperature for 10 min and centrifuged at 12,000 × *g* for 5 min. Supernatants were incubated with ONPG substrate (in 1 mM MgCl_2_, 45 mM β-mercaptoethanol, 0.1 M sodium phosphate pH 7.4) in a 96-well plate at 37 °C for 30 min before adding stop solution (3,3’,5,5’-tetramethylbenzidine, TMB; Thermo Scientific). Absorbance was measured at 405–420 nm using a spectrophotometer (Molecular Devices).

### *In vitro* delivery of Cre-recombinase by CPPs

Cre recombinase was conjugated with CPPs as described for CPP-β-galactosidase conjugates. Recombinant Cre recombinase containing a nuclear localization sequence (NLS) was purchased from Excellgen (Rockville, MD, USA). Fibroblast cells transfected with pCALNL-DsRed were seeded into confocal bottom dishes, grown to 80% confluence, washed twice with serum-free DMEM, and 500 μL of serum-free medium was added. A 100 μL solution containing conjugation buffer, CPPs (1 μM), 25 μg Cre recombinase (5 mg/mL), and 4.8 μg transglutaminase was incubated at 37 °C for 1 h. The resulting conjugates were added to the cells and incubated at 37 °C for 6 h. Cells were washed thrice with serum-free DMEM and analyzed using flow cytometry and confocal laser scanning microscopy.

### *In vivo* distribution of β-galactosidase by CPPs

All experiments with live animals were performed in compliance with the relevant laws and institutional guidelines of Korea Institute of Science and Technology (KIST), and institutional committees have approved the experiments. Five weeks-old BALB/c mice were injected intraperitoneally with 300 μg of CPP-β-galactosidase conjugates in 0.3 mL of PBS. After 2 h, mice were sacrificed and perfused. The main organs (brain, liver, lung, kidney, heart, and spleen) were harvested, washed with PBS, fixed in 0.25% glutaraldehyde and 1.5% formaldehyde for 2 h, and developed in 1 mg/mL X-gal staining solution at 37 °C overnight. For tissue sections, organs were cut into 15 μm sections on a cryomicrotome, fixed, and stained as above. Sections were analyzed using microscopy (Nuance, Olympus; 10 ×). For quantitative enzyme assays, the excised tissues were homogenized under liquid nitrogen and lysed with lysis buffer (250 mM Tris-HCl, pH 7.2, 0.1% Triton X-100) on ice for 30 min. The lysed tissues were centrifuged at 12,000 × *g* for 10 min at 4 °C, and 100 μL of supernatant from each sample with normalized total protein concentration was mixed with 100 μL of assay buffer (200 mM sodium phosphate, pH 7.3, 2 mM MgCl_2_, 100 mM β-mercaptoethanol, 1.33 mg/ml ONPG) and incubated at 37 °C for 30 min. Na_2_CO_3_ (1 M) was added to the reaction mixtures, and absorbance was measured at 420 nm using a microwell plate reader (Molecular Devices).

### Statistical analysis

Two-way analysis of variance (ANOVA) was used to establish the statistical significance of difference in terms of peptide and the effect of fused peptides for each parameter. P-value summary for each comparison with control was inserted in figures (***P < 0.001; *P < 0.05).

## Additional Information

**How to cite this article**: Kim, H.Y. *et al.* Discovery of a non-cationic cell penetrating peptide derived from membrane-interacting human proteins and its potential as a protein delivery carrier. *Sci. Rep.* **5**, 11719; doi: 10.1038/srep11719 (2015).

## Supplementary Material

Supplementary Information

## Figures and Tables

**Table 1:** Synthesized sequences of N-terminus annexin isoforms and the sequences of CPP-PLP conjugates.

Name	Sequence	[M+H]^+^_calc_^a^	[M+H]^+^_obs_^b^	pI	Charge^c^
AA1H	FITC-MAMVSEFLKQ	1685.0	1685.6	5.61	−1
AA2H	FITC-MSTVHEILCK	1662.0	1662.6	8.86	−1
AA3H	FITC-MASIWVGHRG	1614.9	1615.8	10.06	0
AA4H	FITC-MATKGGTVKA	1464.7	1464.9	10.80	1
AA5H	FITC-MAQVLRGTVT	1576.9	1578.9	10.06	0
AA6H	FITC-MAKPAQGAKY	1565.8	1564.2	10.23	1
AA7H	FITC-MSYPGYPPTG	1570.8	1571.9	5.28	−1
AA8H	FITC-MAWWKSWIEQ	1866.2	1865.1	5.61	−1
AA9H	FITC-MSVTGGKMAP	1479.8	1478.9	8.86	0
AA10H	FITC-MFCGDYVQGT	1621.8	1620.9	3.75	−1
AA11H	FITC-MSYPGYPPPP	1606.8	1605.4	5.28	−1
AA13H	FITC-MGNRHAKASS	1559.7	1560.4	11.66	1
TAT_47-58_	FITC-YGRKKRRQRRR	2061.4	2060.1	12.81	7
					
Sequences of protein conjugated CPPs					
Name	Sequence	[M+H]^+^_calc_^†^	[M+H]^+^_obs_^‡^	pI	Charge
PLP	FITC-RRRQQQQQQRRR	2225.5	2226.9	13.20	5
AA3H-PLP	FITC-MASIWVGHRG-RRRQQQQQQRRR	3434.0	3433.8	13.28	7
TAT-PLP	FITC-YGRKKRRQRRR-RRRQQQQQQRRR	3880.0	3878.6	13.74	13

^a^Calculated molecular weight.

^b^Observed molecular weight in MALDI-TOF MS.

^c^At pH 7.4.
