# Supplementary material for: Discovery of a non-cationic cell penetrating peptide derived from membrane-interacting human proteins and its potential as a protein delivery carrier
Source: Sci Rep. 2015 Jun 26;5:11719. doi: 10.1038/srep11719 (PMC4481774; doi:10.1038/srep11719)
Supplement: Supplementary Information [file srep11719-s1.pdf]

Discovery of a non-cationic cell penetrating peptide derived from membrane-interacting human proteins and its potential as a protein delivery carrier

Hyo Young Kim[a], Soo Young Yum[c], Goo Jang[c], and Dae-Ro Ahn[a,b]*

[a] Center for Theragnosis, Biomedical Research Institute, Korea Institute of Science and Technology (KIST), Hwarangno 14-gil 5, Seongbuk-gu, Seoul 136-791, Republic of Korea

[b] Department of Biological Chemistry, Korea University of Science and Technology, KIST campus, Republic of Korea

[c] Laboratory of Theragenology and biotechnology, Department of Veterinary Clinical Science, College of Veterinary Medicine, Seoul National University, 1 Gwanak-ro, Gwanak-gu, Seoul, Republic of Korea, 151-742

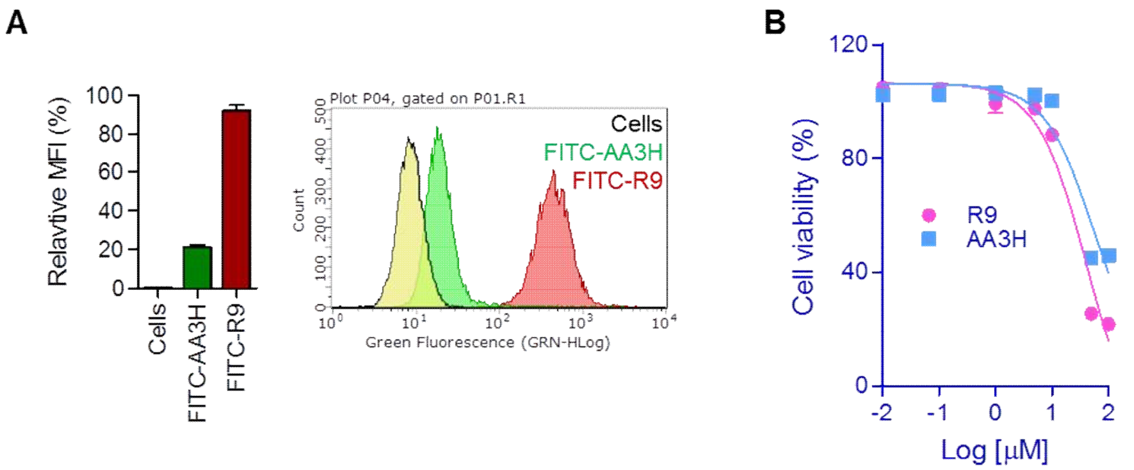

Supplementary Fig. S1. Comparison of cellular uptake (A) and cytotoxicity (B) of CPPs.

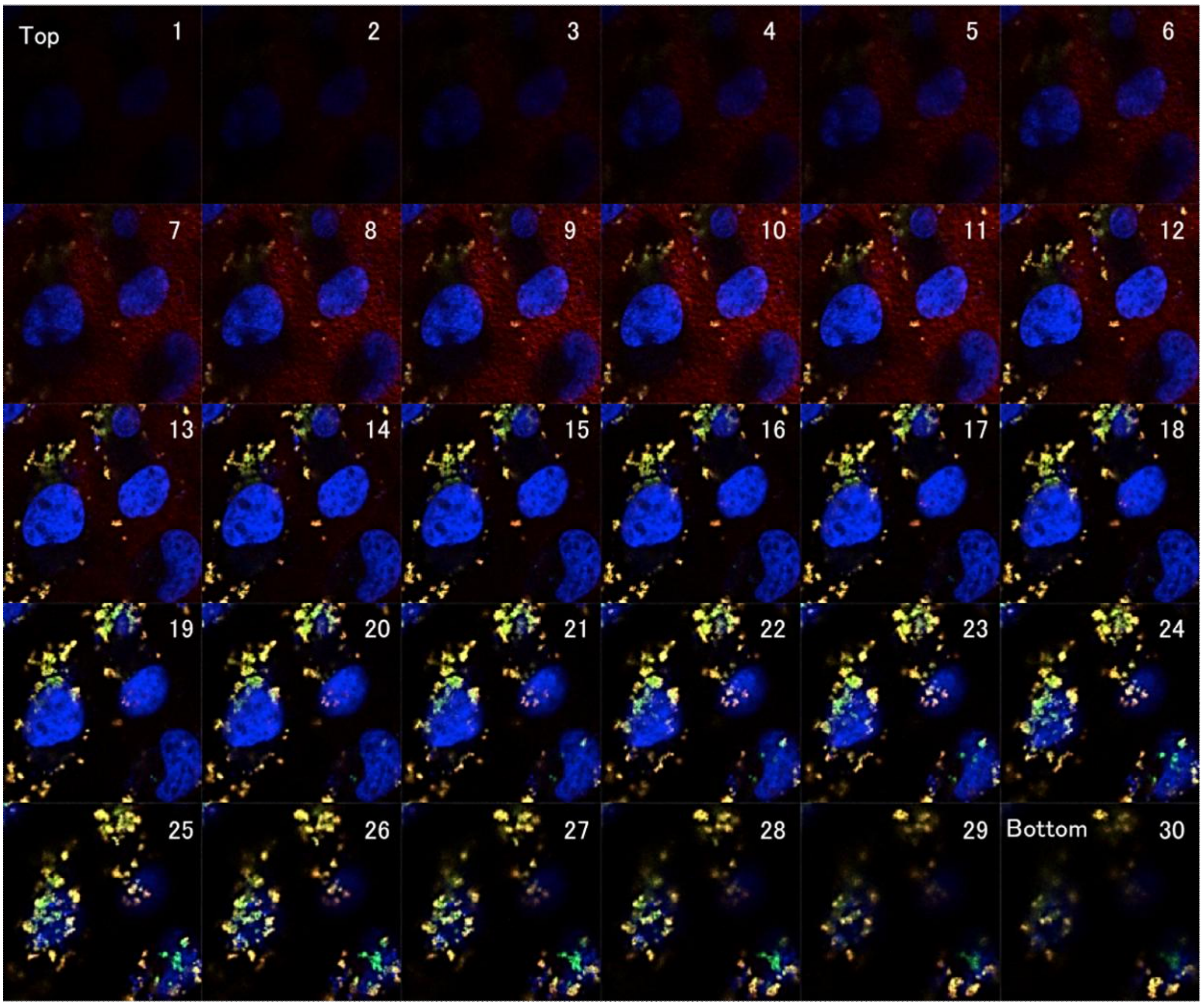

Supplementary Fig. S2. Z-stack confocal microscopic images of AA3H-treated HeLa cells.

Liposome experiment. Phospholipids dissolved in chloroform (L-alpha-phosphatidylcholine: PC, L-alpha-phosphatidylserine: PS, and L-alpha-phosphatidylethanolamine: PE) were purchased from Avanti Polar Lipids. Liposomes were prepared by following a reported procedure[1]. After preparation of phospholipid mixtures with appropriate molar ratios (PC/PS: 8/2 and PC/PE/PS: 4/4/2), the mixtures were dried by removing the solvent. The residues were dissolved in HBS (20 mM HEPES/NaOH, pH 7.5, 100 mM NaCl, 0.02% (w/v) sodium azide) and vortexed to completely resuspend phospholipids. Then, the suspension was sonicated until to have clear solution. The prepared vesicles was incubated with AA3H-CPP (FITC labelled) for 4 h at room temperature and the mixture was centrifuged at 16,000×g for 10 min at 4°C before flow cytometric analysis.

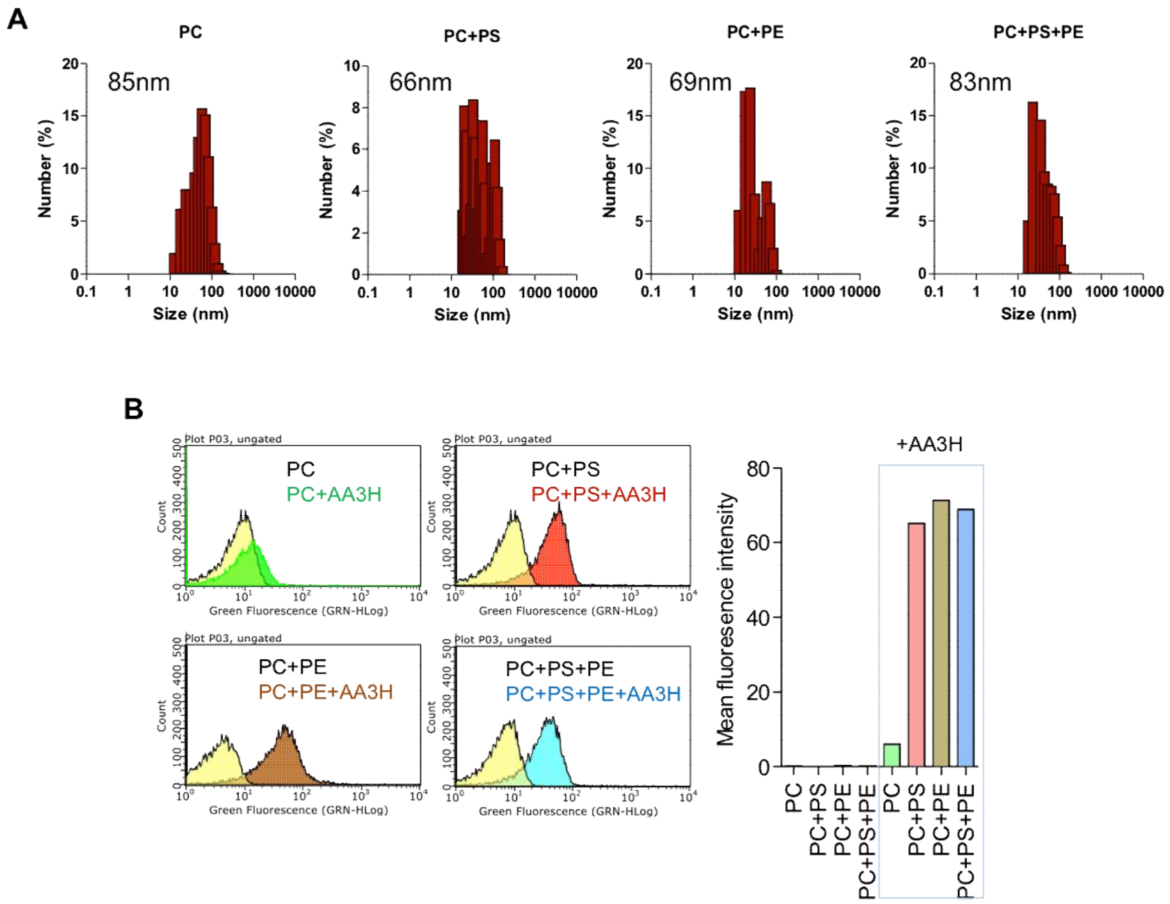

Supplementary Fig. S3. (A) Sizes of liposomes determined by dynamic light scattering. (B) Flow cytometric analysis on the interaction between FITC-labeled AA3H and liposomes.

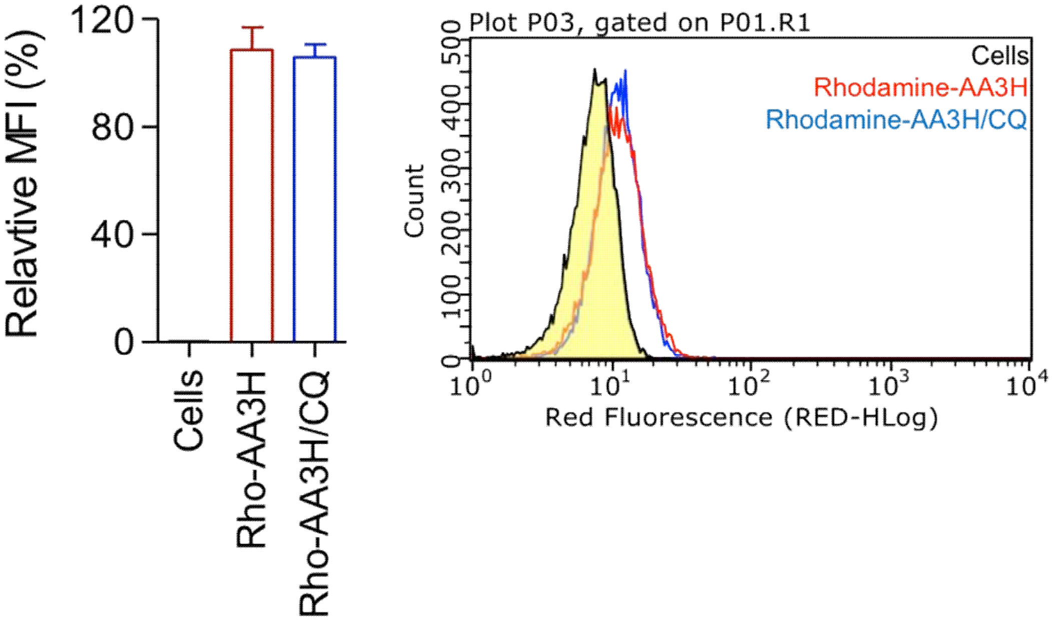

Supplementary Fig. S4. Flow cytometric analysis of cells treated with tetramethylrhodamine-AA3H (Rho-AA3H) in the presence and absence of chloroquin (CQ).

RFP delivery by CPPs. The conjugation reaction was carried out by the same manner employed for preparation of CPP-β-galactosidase conjugates. After the reaction, unconjugated protein and the possible TAT dimer were removed by using 40K zeba column (Thermo scientific, IL, USA). The number of peptides conjugated per one RFP molecule was determined by measuring absorbance of the conjugates at 490 nm and 550 nm respectively. HeLa cells were treated with CPP-RFPs for 4 h and examined by using a flow cytometer for quantitative analysis of intracellular uptake of RFP.

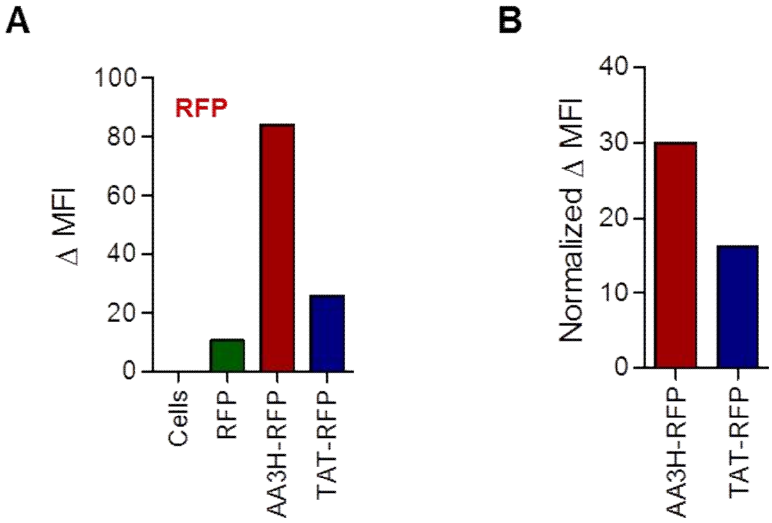

Supplementary Fig. S5. (A) Flow cytometric analysis on uptake of RFP into cells treated with RFP, AA3H-RFP, and TAT-RFP. (B) Normalized uptake efficiency of RFP based on the number of conjugated peptides on the protein has been determined by Δ MFI/ the number of peptides.

1. Akbarzadeh, A. et al. Liposome: classification, preparation, and applications. *Nanoscale Res Lett* **8**, 102, 10.1186/1556-276X-8-102 (2013).
